# Tunicates push the limits of animal evo-devo

**DOI:** 10.1186/1741-7007-9-3

**Published:** 2011-01-20

**Authors:** David EK Ferrier

**Affiliations:** 1 The Scottish Oceans Institute, University of St Andrews, East Sands, St Andrews, KY16 8LB, UK

## Abstract

The phylum to which humans belong, Chordata, takes its name from one of the major shared derived features of the group, the notochord. All chordates have a notochord, at least during embryogenesis, and there is little doubt about notochord homology at the morphological level. A study in *BMC Evolutionary Biology *now shows that there is greater variability in the molecular genetics underlying notochord development than previously appreciated.

See research article: http://www.biomedcentral.com/1471-2148/11/21

## Commentary

**Figure 1:**
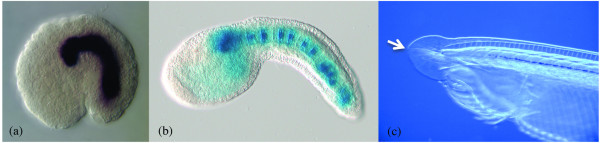
**Invertebrate chordate notochords**. **(a) **Expression of the gene *brachyury *(*Odi-T*) in the developing notochord of *Oikopleura dioica *(image courtesy of Cristian Cañestro and Susan Bassham). **(b) **The *Ciona intestinalis *notochord visualized with a *brachyury *LacZ reporter gene (image courtesy of Peter Osborne). **(c) **The head of a juvenile amphioxus (*Branchiostoma lanceolatum*), with the notochord distinguishable up to the anterior-most tip of the animal's head and indicated by the arrow (image by the author).

The phylum Chordata includes the Cephalochordata (amphioxus), the Urochordata (tunicates such as sea squirts, salps and larvaceans) and the Vertebrata (fish, amphibians, reptiles, birds and mammals), and a defining feature of the chordates is the presence of a notochord in at least some stage of life. The notochord is a stiff rod of tissue located ventral to the neural tube. Some chordates retain the notochord throughout their lives, whereas in others it is only present during embryogenesis and larval life (Figure 1). For example, the notochord is a permanent feature of amphioxus (for example, *Branchiostoma lanceolatum*) and the larvacean *Oikopleura dioica*, but is lost at metamorphosis in the sea squirt *Ciona intestinalis *and is replaced in vertebrates by the vertebral column after embryogenesis. The notochord is a source of important embryonic developmental signals [1], with a role in coordinating the development of the notochord, central nervous system (CNS) and mesoderm. It also provides a mechanical supporting function - giving the body some rigidity that the axial musculature can act against.

## 'Fuzzy' homology

Underlying the variability in the roles and morphogenesis of the notochord across the chordates is a core conserved feature: the expression of the gene *brachyury *(*bra*) and its apparent involvement in notochord specification throughout the phylum [2] (Figure 1). Notwithstanding the important discussions of what constitutes homology, and the need to be clear about the level at which one is analyzing homology [3], *bra *expression in the chordate notochord is a good example of a homologous gene with a homologous function in a homologous morphological character, the notochord, with the added importance that this character is one of the distinguishing features of our own phylum. A study published in *BMC Evolutionary Biology *by Kugler *et al*. [4], which compares the targets of *bra *in *Ciona *and *Oikopleura*, throws new light on the extent of evolutionary divergence of notochord development.

With the burgeoning amounts of gene sequence and expression data now available it is becoming feasible to go beyond the matching of individual homologous genes to homologous morphologies and instead build gene networks and profiles for developmental or morphological characters. This permits stronger tests of homology (as opposed to convergent evolution by the cooption of single homologous genes) as well as potentially revealing the evolutionary dynamics of the character of interest; in other words, what is the genetic 'essence' of a character and how great is the evolutionary lability of a character in terms of the molecular aspects of its construction?

As we start constructing gene networks for the development of morphological features (such as arthropod - or even other bilaterian - segments [5], bilaterian nervous systems [6] and now notochords [4]), the need and the ability to distinguish between homologous, convergent or superficially similar molecular networks are becoming more apparent and more feasible. Networks clearly do not have to be exactly the same, with entirely identical members, to be homologous. But how quickly do they change from a common ancestral state, and how much variability is tolerated while still producing equivalent, homologous functions or morphologies? Rates of evolution often differ between lineages (for example, slow-versus fast-clock lineages), and so there is an element of making these judgments on a case-by-case basis - the messy nature of biology. But these issues go to the heart of biology, as biologists do not investigate processes and phenomena with the view that they are so individual and specialized that they cannot tell us anything about other biological systems. Instead, we are always on the lookout for general principles and wider applicability. Studying a process in one species, we hope, will tell us general things about the process, the mechanisms and the biology of many species. This is the comparative method that underpins all biology, and it stands or falls on just how similar or different, or how well conserved or divergent and unique, the various species are.

## How to be different while staying the same

In the context of notochord development, *Oikopleura *can now be seen to probably be pushing the limits of what is possible in the evolutionary lability of homologous developmental gene networks or profiles. Kugler *et al. *[4] began with a list of 50 genes that are known to be *bra *targets in *Ciona intestinalis *and looked for these genes in the larvacean urochordate *Oikopleura dioica*. Of these 50 *Ciona *genes almost half (24 of 50) are missing from the *Oikopleura *genome. Of the remaining genes, some of which have undergone lineage-specific duplications, Kugler *et al*. find that only 13 out of 28 have clear expression in the notochord in *Oikopleura*, while a further seven have diffuse expression throughout the tail (and so potentially also have some expression in the notochord). Clearly, there has been dramatic evolution downstream of *bra *in the urochordate notochord.

The search for *bra *targets in *Oikopleura *was greatly facilitated by the availability of a whole-genome sequence [7]. The value of genome sequences for determining gene repertoires, and having reasonable confidence about gene paralogy (where genes are homologous by virtue of a duplication rather than speciation event) and gene presence/loss, is crucial to developmental genomics analyses, such as that for the notochord. In addition, whole-genome sequences are now permitting the recognition of lineages that are more or less derived from ancestral states (that is, those lineages that have conserved many similarities to the ancestor versus those that have undergone many evolutionary changes). For example, the amphioxus genome is less derived from the ancestral chordate genome than are urochordate genomes [7,8]; the genomes of urochordates are evolving more rapidly than that of amphioxus and can reveal more about the extent to which an animal species can be molecularly different from its relatives while retaining the essential features that make it a member of a particular group. *Oikopleura *and *Ciona *are some of the clearest examples of how to be different while staying the same.

*Oikopleura *has an amazingly fast life cycle for an animal - only 4 days at 20°C - which is approaching the generation time of some bacteria. Many *O. dioica *genes are also transcribed within operons, analogous to the operons of bacteria, with 1,761 operons containing up to 11 genes per operon being predicted in *O. dioica *[7]. The *Oikopleura *genome is an extreme case of animal genome malleability, with negligible synteny (conservation of gene linkage and neighborhoods) to other animal genomes [7], in stark contrast to the extensive ancient synteny observed elsewhere - for example, between sea anemones and humans [9].

The extremely rapid life cycle of *Oikopleura *will clearly have some effect on genome evolution, as a result of the relatively high number of generations produced over a given time period. But a crucial question is whether this high number of generations, and the consequent greater opportunity for inherited mutations (rearrangements, gene losses and duplications, and intron changes), is the sole explanation for the oddity of the *Oikopleura *genome and the extremely derived condition of gene profiles such as that of the notochord. An alternative explanation could be that the very short life cycle has required the evolution of very different ways of developing and functioning at the molecular level, such that constraints on the genome organization and content that are present elsewhere in the animal kingdom no longer apply in *Oikopleura*. In other words, comparing the range of mutations occurring over a certain number of generations in *Oikopleura *with those in amphioxus, say, over the same number of generations, would reveal a much higher number of mutations in *Oikopleura*. The underlying biology of these two organisms with regard to the development and maintenance of genome organization may well be fundamentally different. For example, if one could suddenly raise the mutation rate of amphioxus to that of *Oikopleura*, this amphioxus population would die because its underlying biology has not been pushed into such unusual and derived ways of doing things during evolution as has happened for the almost 'bacterially fast' reproducing *Oikopleura*. Some animals (*Oikopleura*) are clearly more derived than others (amphioxus), but have they had to invent new biology, or lose the constraints of old biology, in order to become so unusual?

Intriguingly, this derived nature of *O. dioica *is not restricted to this species within the urochordates, although *O. dioica *is the most extreme case of derivation characterized so far. The evolutionary journey towards the derived genome and development of this larvacean has been traveled to a lesser degree by other urochordates, such as *Ciona*, which also has relatively rapid rates of evolution of gene sequence, content and organization, and seems to have largely dispensed with the embryo-patterning roles of ancient developmental control genes such as the Hox genes, which have important developmental control functions elsewhere across the animal kingdom [10]. Interestingly, the organization of the Hox genes in an ordered cluster has also been lost in urochordates, with *Oikopleura *representing the most derived, dispersed set of Hox genes known [11].

The expression studies of Kugler *et al. *[4] highlight the potential of the notochord system as a model for identifying functional links between genes and building gene networks of notochord development in different species. This needs to go hand-in-hand with wider taxonomic comparisons across the chordates (and beyond) to enable precise deduction of the extent of conservation or divergence of the notochord-building networks and whether there is a general notochord 'kernel' for the chordates beyond the expression of *brachyury*. These are early days for such evolutionary developmental genomics, with much work to be done to understand not just the development of the notochord in one or two model chordates, but across the chordates as a whole, so that the evolutionary dynamics can be properly understood. To reveal the scope for development of homologous characters via different, divergent (rather than conserved) genetic networks (the so-called 'inverse paradox' in evo-devo [12]), taxonomically wide sampling is essential, and goes to the heart of our understanding of homology and the power of the comparative method. The notochord, as an evolutionary novelty that distinguishes our own phylum, is a prime candidate for such an effort.

## References

[B1] Stemple DL Structure and function of the notochord: an essential organ for chordate development Development 2005 132 2503 2512 10.1242/dev.01812 15890825

[B2] Bassham S Postlethwait J *Brachyury *(*T*) expression in embryos of a larvacean urochordate, *Oikopleura dioica*, and the ancestral role of *T* Dev Biol 2000 220 322 332 10.1006/dbio.2000.9647 10753519

[B3] Wagner GP The developmental genetics of homology Nat Rev Genet 2007 8 473 479 10.1038/nrg2099 17486120

[B4] Kugler JE Kerner P Bouquet JM Jiang D Di Gregorio A Evolutionary changes in the notochord genetic toolkit: a comparative analysis of notochord genes in the ascidian *Ciona *and the larvacean *Oikopleura* BMC Evol Biol 2011 11 21 10.1186/1471-2148-11-21 PMC3034685 21251251

[B5] Dray N Tessmar-Raible K Le Gouar M Vibert L Christodoulou F Schipany K Guillou A Zantke J Snyman H Béhague J Vervoort M Arendt D Balavoine G Hedgehog signaling regulates segment formation in the annelid *Platynereis* Science 2010 329 339 342 10.1126/science.1188913 20647470 PMC3182550

[B6] Denes AS Jekely G Steinmetz PR Raible F Snyman H Prud'homme B Ferrier DEK Balavoine G Arendt D Molecular architecture of annelid nerve cord supports common origin of nervous system centralization in bilateria Cell 2007 129 277 288 10.1016/j.cell.2007.02.040 17448990

[B7] Denoeud F Henriet S Mungpakdee S Aury JM Da Silva C Brinkmann H Mikhaleva J Olsen LC Jubin C Cañestro C Bouquet JM Danks G Poulain J Campsteijn C Adamski M Cross I Yadetie F Muffato M Louis A Butcher S Tsagkogeorga G Konrad A Singh S Jensen MF Cong EH Eikeseth-Otteraa H Noel B Anthouard V Porcel BM Kachouri-Lafond R Plasticity of animal genome architecture unmasked by rapid evolution of a pelagic tunicate Science 2010 330 1381 1385 10.1126/science.1194167 21097902 PMC3760481

[B8] Putnam NH Butts T Ferrier DE Furlong RF Hellsten U Kawashima T Robinson-Rechavi M Shoguchi E Terry A Yu JK Benito-Gutiérrez EL Dubchak I Garcia-Fernàndez J Gibson-Brown JJ Grigoriev IV Horton AC de Jong PJ Jurka J Kapitonov VV Kohara Y Kuroki Y Lindquist E Lucas S Osoegawa K Pennacchio LA Salamov AA Satou Y Sauka-Spengler T Schmutz J Shin-I T The amphioxus genome and the evolution of the chordate karyotype Nature 2008 453 1064 1071 10.1038/nature06967 18563158

[B9] Putnam NH Srivastava M Hellsten U Dirks B Chapman J Salamov A Terry A Shapiro H Lindquist E Kapitonov VV Jurka J Genikhovich G Grigoriev IV Lucas SM Steele RE Finnerty JR Technau U Martindale MQ Rokhsar DS Sea anemone genome reveals ancestral eumetazoan gene repertoire and genomic organization Science 2007 317 86 94 10.1126/science.1139158 17615350

[B10] Ikuta T Satoh N Saiga H Limited functions of Hox genes in the larval development of the ascidian *Ciona intestinalis* Development 2010 137 1505 1513 10.1242/dev.046938 20335361

[B11] Seo HC Edvardsen RB Maeland AD Bjordal M Jensen MF Hansen A Flaat M Weissenbach J Lehrach H Wincker P Reinhardt R Chourrout D Hox cluster disintegration with persistent anteroposterior order of expression in *Oikopleura dioica* Nature 2004 431 67 71 10.1038/nature02709 15343333

[B12] Canestro C Yokoi H Postlethwait JH Evolutionary developmental biology and genomics Nat Rev Genet 2007 8 932 942 10.1038/nrg2226 18007650

